# UP Finder: A COBRA toolbox extension for identifying gene overexpression strategies for targeted overproduction

**DOI:** 10.1016/j.meteno.2017.08.001

**Published:** 2017-08-16

**Authors:** Xi Wang, Liang Yu, Shulin Chen

**Affiliations:** Department of Biological Systems Engineering, Washington State University, Pullman, WA 99164, USA

**Keywords:** COBRA toolbox, Gene overexpression strategy, Metabolic engineering, Targeted overproduction, Rational pathway design

## Abstract

Overexpression of key genes is a basic strategy for overproducing target products via metabolic engineering. Traditionally, identifying those key genes/pathways largely relies on the knowledge of biochemistry and bioinformatics. In this study, a modeling tool named UP Finder was developed to facilitate the rapid identification of gene overexpression strategies. It was based on the COBRA toolbox under MATLAB environment. All the key gene/pathway targets are identified in one click after simply loading a Systems Biology Markup Language model and specifying a metabolite as the targeted product. The outputs are also quantitatively ranked to show the preference for determining overexpression strategies in pathway design. Analysis examples for overproducing lycopene precursor in *Escherichia coli* and fatty acyl-ACP in the cyanobacterium *Synechocystis* sp. PCC 6803 by the UP Finder showed high degree of agreement with the reported key genes in the literatures.

## Highlights

• A modeling interface is developed for identifying gene overexpression targets.
• It evaluates the yield contribution for each overexpression candidate.
• The outputs are ranked to show the preference for determining strategies.

## Graphical abstract

**Figure:**
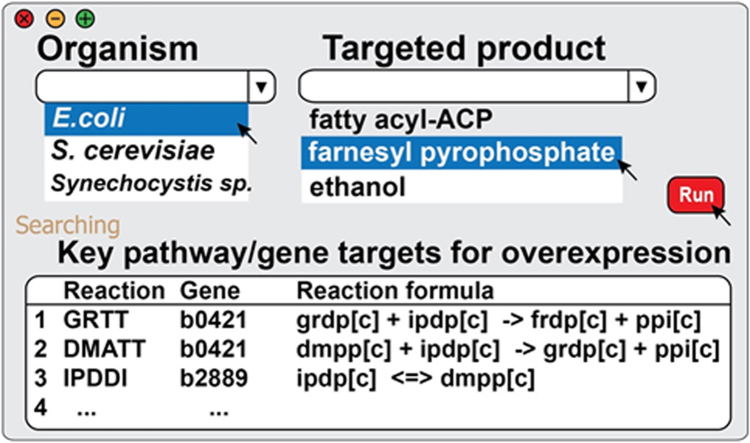

## 1 Introduction

Engineering microorganisms to overproduce interested products is an important practice in metabolic engineering. In the successful examples, overexpressing key genes of metabolic pathways is a widely used strategy for achieving overproduction (Ajikumar et al., 2010, Alper et al., 2005). The purpose is to up-regulate the flux for substrate synthesis or to intensify the shunt at key metabolic nodes toward an improved flux to targeted metabolites. Since the overproduction of natively synthesized metabolites is usually achieved by genetically manipulating metabolic pathways, identifying the key pathways and gene targets is a key step to determine gene overexpression strategies for consequential manipulations. Traditionally, completion of such tasks was largely relying on the experience of metabolic pathways and enzymatic kinetics. However, with the increasing practices of metabolic engineering in overproducing fuels, chemicals and natural products (Stephanopoulos, 2012), empirical predictions have been hardly satisfying the analysis of sophisticated pathways, such as the multiple-repeated pathways in fatty acid synthesis and the rarely explored secondary metabolite biosynthesis. Therefore, it is critical to establish a standard procedure for identifying gene overexpression strategies.

The rapid advances of constraint-based models provide the possibilities for quantitative evaluation of cellular metabolism (Bordbar et al., 2015, Kauffman et al., 2003), allowing to develop the standard method for rational pathway design. According to the annotated genome information, the reconstructed constraint-based models could represent the current knowledge of full metabolic reactions and their associated genes for an organism. With those constraint-based models, algorithms such as flux balance analysis (FBA) were developed to perform the *in silico* analysis of metabolic fluxes (Orth et al., 2010). Relying on the principle of mathematical optimization and mass balance, metabolic fluxes can be simulated within determined constraints. Such efforts have advanced the development of modeling approaches such as OptKnock (Burgard et al., 2003) that facilitates the procedures for identifying gene targets and pathway design.

Unlike gene knockout based simulation, *in silico* identifying gene overexpression targets has more uncertainties to be experimentally verified because of the difficulties for exactly manipulating fluxes to certain values. To overcome this challenge, methodologies have been developed for simulating gene overexpression, such as OptForce (Ranganathan et al., 2010) and FSEOF (Choi et al., 2010), as well as their derivatives (Chowdhury et al., 2014, Park et al., 2012). By using enforced flux and flux variability analysis, gene targets with desired up-regulation were successfully simulated and experimentally verified. However, those overexpressed gene targets were mostly identified to coordinate with additional manipulations (e.g. knockouts or down-regulation), whereby overexpressing some targets such as targets in glycolysis may not always independently contribute to an overproduction. Therefore, it is important to know the contribution of each candidate targets toward the theoretical maximum yield to fulfill the growing needs on customized pathway design. In addition, most current modeling methods still require specific programming skills that restricts the access for biologists and broad users. It is highly desirable to develop the software platform that can bridge the technical gap between computational modeling and bench works. In this paper, we present a software package, UP Finder that facilitates the identification of gene overexpression strategies for the metabolic engineering of targeted overproduction. It highlighted the quantitative evaluation for each overexpression candidate on yield contribution. The graphical user interface of the UP Finder also provided easier access for broad users. Two typical examples in metabolic engineering that lycopene precursor and fatty acyl-ACP overproduction were used to evaluate feasibilities of the UP Finder for analyzing biosynthesis pathways of natural products and biofuels. The identified gene targets by the UP Finder showed high degree of agreement with the reported key genes in the literatures.

## 2 Materials and methods

### 2.1 Models and FBA

The metabolic reconstructed model of *Escherichia coli* iJO1366 (Orth et al., 2011) was used for analyzing gene overexpression strategies in lycopene precursor overproduction. And the reconstructed model of *Synechocystis* sp. PCC 6803 iJN678 (Nogales et al., 2012) was used for the analysis of fatty acyl-ACP overproduction.

FBA was used for all model analysis. For wild-type model, the defaulted biomass formulation was used as the objective function for maximizing cell growth. For theoretical maximum yield model, the targeted product was used as the objective function for maximizing the production of targeted product, such as farnesyl pyrophosphate and fatty acyl-ACP discussed in Results.

All computation was performed on Mac OS × 10.6.8, 1.86 GHz Inter Core 2 Duo Processor, 2 GB 1067 MHz DDR3 Memory. COBRA toolbox v2.0.5 was added to the path of MATLAB_R2012b, including SBML Toolbox_4.1.0 bundled in the package. libSBML_5.7.0 was installed to access the Systems Biology Makeup Language. Gurobi_5.1.0 was used as the LP solver.

### 2.2 Definition of parameters

The parameter *flux*_*wt*_ represents wild-type flux that is the flux solution of the wild-type model, and *flux*_*opt*_ represents the optimum flux that is the flux solution of the theoretical maximum yield model. The up-regulation ratio (*Ratio*) is defined as the ratio of *flux*_*opt*_ to *flux*_*wt*_ of a reaction (*Ratio* = *flux*_*opt*_ / *flux*_*wt*_). And the *Yield* is simulated product yield of the targeted product by using *flux*_*opt*_ of a reaction as the constraint, in which maximizing cell growth is the objective function.

### 2.3 Development of the UP Finder

**Fig. 1:**
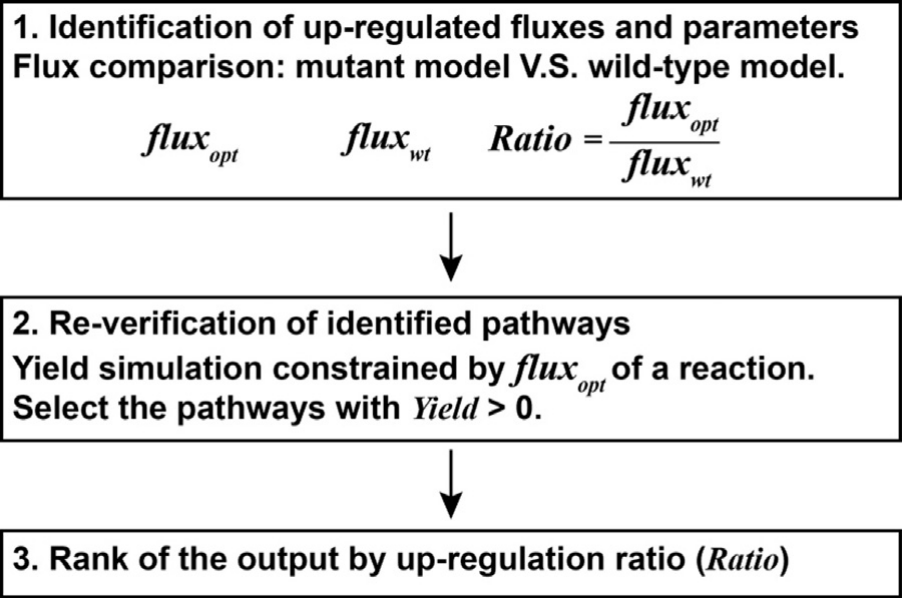
The working procedure of the UP Finder. Mutant model, the overproducing metabolic network, the model with flux distribution under the theoretical maximum yield conditions (flux distribution for reaching theoretical maximum yield of a metabolite); *flux*_*wt*_, wild-type flux, which is the flux distribution of the wild-type conditions; *flux*_*opt*_, optimum flux, which is the flux distribution of the theoretical maximum yield conditions; *Ratio*, up-regulation ratio, which is the ratio of the optimum flux to the wild-type flux of a reaction; *Yield*, simulated yield of the targeted product by using the optimum flux of a reaction as the constraint.

UP Finder is an interfacial modeling tool based on the COBRA toolbox in MATLAB, which is developed by the MATLAB Graphical User Interface Development Environment (GUIDE). It is used to identify all the key gene targets for overexpression that directly related to the overproduction of a metabolite in a microorganism. The working procedure of the UP Finder is composed of following steps (Fig. 1):

(1) Identification of up-regulated fluxes. The main concept is to compare the flux distributions between the wild-type and overproducing metabolic networks by calculating theoretical maximum yield of a targeted product. Thus, up-regulated fluxes and their associated pathways (termed as up-regulated pathways in this study) can be identified through this comparison.
(2) Re-verification of identified pathways. Since not all the identified pathways from Step (1) are directly related to the overproduction, a re-verification is necessary to filter the low-relevant targets. For these identified pathways, their fluxes under overproducing networks were considered as the optimum fluxes (*flux*_*opt*_) to achieve theoretical maximum yield of the product. The simulated product yields (*Yield*) constrained by each *flux*_*opt*_ for the wild-type network were used to evaluate the best contribution of each up-regulated pathway toward overproduction. Pathways with *Yield* > 0 are considered as the key targets that directly lead to the overproduction.
(3) Rank of the output. The output of the UP Finder is the abbreviated reaction names of the selected key pathways in Step (2). A termed parameter, *Ratio,* which is the ratio of each *flux*_*opt*_ over their associated wild-type fluxes (*flux*_*wt*_) was used for ranking the output from high to low. Because *Ratio* reflects the up-regulated level for each reaction, the one with the highest *Ratio* value suggests the highest preference when considering gene overexpression in engineering of the targeted overproduction.

### 2.4 Implementation

**Fig. 2:**
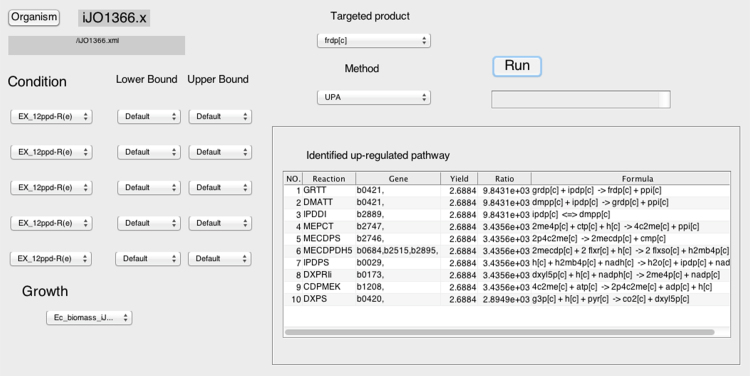
The interface of the UP Finder. The interface contains 5 major functional units, including the *Organism*, *Condition*/*Growth*, *Targeted product*, *Method* and *Output. Condition* is the exchange reactions of SBML models with their constraints, and *Growth* indicates the specific biomass objective function (e.g. autotrophic or heterotrophic growth for *Synechocystis* sp. PCC 6803). *Method* contains two computational methods: UPA (up-regulated pathway analysis) and FBA (flux balance analysis).

Through the interface of the UP Finder, after loading a Systems Biology Markup Language (SBML) model in the *Organism* item, all metabolites included in this model will be shown in the *Targeted product* item. Users can simply specify one metabolite as the target for overproduction. By choosing UPA in the *Method* option and running the program, a list of reaction names presented with their associated genes, reaction formulas, *Yield* and *Ratio* values will be returned as the output. All computation is based on the COBRA toolbox and MATLAB, and all optimization uses FBA for the solutions (Orth et al., 2010, Schellenberger et al., 2011). Initializing the COBRA toolbox is necessary in MATLAB before loading SBML models. The default uptake and growth constraints of the reconstructed model are used for the analysis. Users can also adjust the uptake and growth conditions to simulate metabolisms with special requirements. In addition, the UP Finder integrates FBA optimization in the *Method* option, which allows the basic function for computing growth rates under different conditions (Fig. 2). The UP Finder is freely available from GitHub (https://github.com/MEpathway/UP-Finder.git).

## 3 Results

### 3.1 Example 1. Lycopene precursor overproduction in *E. coli* (iJO1366.xml)

**Fig. 3:**
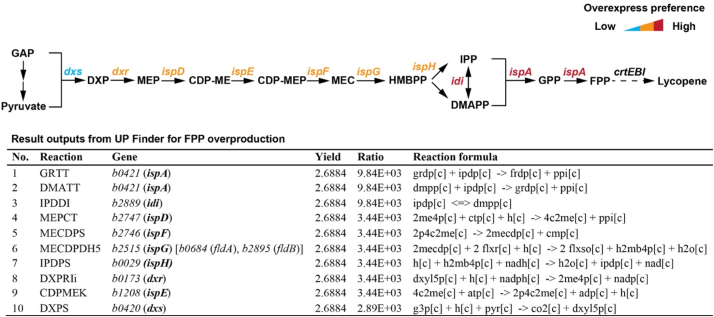
Analysis results from the UP Finder for farnesyl pyrophosphate (FPP) overproduction in *E. coli.* Identified gene targets are also shown in the metabolic pathway of FPP biosynthesis presented with gene overexpression preference toward FPP overproduction. GAP, glyceraldehyde-3-phosphate; DXP, 1-deoxy-D-xylulose 5-phosphate; MEP, 2-C-methyl-D-erythritol 4-phosphate; CDP-ME, 4-diphosphocytidyl-2-C-methyl-D-erythritol; CDP-MEP, 4-diphosphocytidyl-2C-methyl-D-erythritol-2-phosphate; MEC, 2C-methyl-D-erythritol-2,4-cyclodiphosphate; HMBPP, (E)−4-hydroxy-3-methylbut-2-enyl-diphosphate; IPP, isopentenyl diphosphate; DMAPP, dimethylallyl diphosphate; GPP, geranyl pyrophosphate; FPP, farnesyl pyrophosphate. *dxs*, 1-deoxy-D-xylulose-5-phosphate synthase; *dxr*, 1-deoxy-D-xylulose reductoisomerase; *ispD*, 2-C-methyl-D-erythritol 4-phosphate cytidylyltransferase; *ispE*, 4-(cytidine 5′-diphospho)−2-C-methyl-D-erythritol kinase; *ispF*, 2-C-methyl-D-erythritol 2,4-cyclodiphosphate synthase; *ispG*, 2C-methyl-D-erythritol 2,4 cyclodiphosphate dehydratase; *ispH*, 1-hydroxy-2-methyl-2-(*E*)-butenyl 4-diphosphate reductase; *idi*, isopentenyl-diphosphate D-isomerase; *ispA*, geranyltranstransferase (farnesyl diphosphate synthase); *crtE*, GGPP synthase, *crtB*, phytoene synthase; *crtI*, phytoene desaturase. See Supplementary Table S1–S2 for the abbreviations of reaction and metabolite names shown in the UP Finder results.

As an important isoprenoid, lycopene overproduction is a textbook example in metabolic engineering. In *E. coli*, overproducing farnesyl pyrophosphate (FPP) is critical for increasing the product yield of lycopene because FPP is the native precursor in lycopene biosynthesis (Alper et al., 2005). Herein, we presented the analysis of FPP (frdp[*c*]) as the *Targeted product* for overproduction in the UP Finder (Fig. 2). By loading the constraint-based model of *E. coli* iJO1366 in the *Organism* item, the output presented key pathway/gene targets for overexpression toward FPP overproduction in *E. coli*. As shown in Fig. 3, ten metabolic reactions (9 genes involved) were identified as the key pathways for FPP overproduction. The identified reactions were ordered by their associated *Ratio* values, which can be used as a quantitative evaluation for the overexpression preference. According to this ranked preference for gene overexpression, it was observed that the higher preferred gene targets showed the closer metabolic distance to the targeted product (FPP), which represented the higher relevance to the related overproduction. Compared with the reported key genes for FPP/lycopene overproduction in the literatures (Wang et al., 2009), results from the UP Finder identified all 9 key genes in isoprenoid biosynthesis that directly related to FPP overproduction, showing 100% identity. Also, comparing to the key genes identified by FSEOF (Choi et al., 2010), those gene targets in central carbon metabolic metabolism were not included in the results of UP Finder (Table 1).

**Table 1:** Comparison of identified gene targets for overproducing lycopene precursor (farnesyl pyrophosphate, FPP) in *E. coli*.

Reported key genes (Wang et al., 2009)	Identified key genes by UP Finder	Identified key genes by FSEOF (Choi et al., 2010)
*dxs*	*dxs*	*dxs*
*dxr*	*dxr*	*dxr*
*ispD*	*ispD*	*ispD*
*ispE*	*ispE*	*ispE*
*ispF*	*ispF*	*ispF*
*ispG*	*ispG*	*ispG*
*ispH*	*ispH*	*ispH*
*idi*	*idi*	*idi*
*ispA*	*ispA*	*ispA*
		*pgi*
		*pfkAB*
		*fbaA*
		*tpiA*
		*gltA*
		*acnAB*
		*icdA*
		*sucAB*
		*sucCD*
		*sdhABCD*
		*fumAB*
		*mdh*

### 3.2 Example 2. Fatty acyl-ACP pool overproduction in the cyanobacterium *Synechocystis* sp. PCC 6803 (iJN678.xml)

**Fig. 4:**
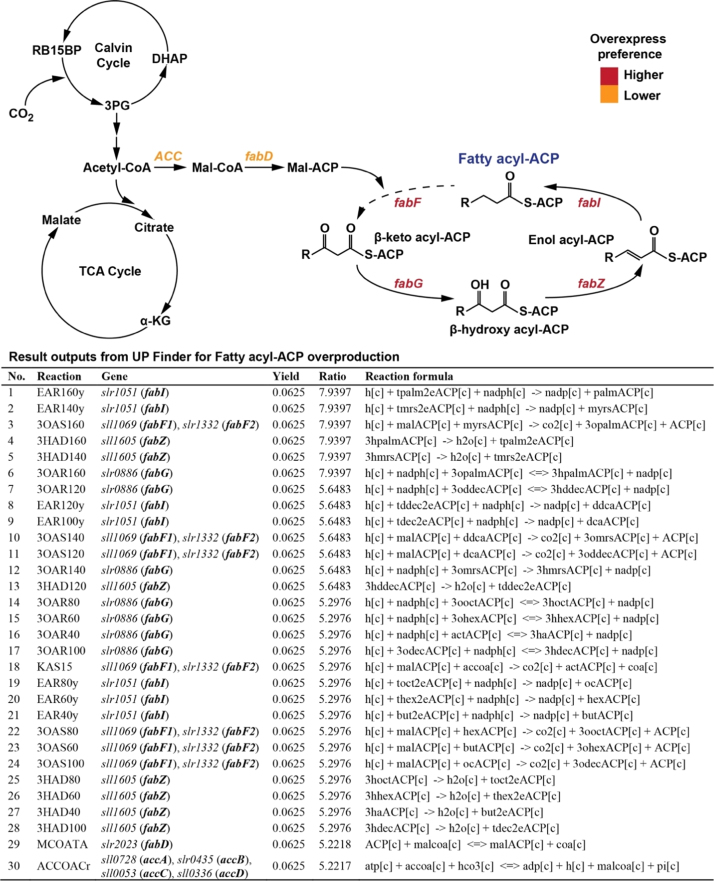
Analysis results from the UP Finder for fatty acyl-ACP (palmitoyl-ACP) overproduction in *Synechocystis* sp. PCC 6803. Identified gene targets are also shown in the metabolic pathway of fatty acyl-ACP biosynthesis presented with gene overexpression preference toward fatty acyl-ACP overproduction. RB15BP, ribulose 1,5-bisphosphate; 3PG, 3-phosphoglycerate; α-KG, α-ketoglutarate; Mal-CoA, Malonyl-CoA; Mal-ACP, Malonyl-ACP. See Supplementary Table S1–S2 for the abbreviations of reaction and metabolite names shown in the UP Finder results.

Directly converting CO_2_ into biofuels is regarded as a promising strategy for producing carbon-neutral renewable energy (Atsumi et al., 2009). Fatty acyl-ACP is the important precursor for the biosynthesis of fatty-acid based biofuel molecules, such as free fatty acids, fatty alcohols and alkanes (Liu et al., 2011). Herein, gene overexpression targets were analyzed for overproducing fatty acyl-ACP in the cyanobacterium *Synechocystis* sp. PCC 6803. As the dominant component of fatty acyl-ACP, palmitoyl-ACP (C16:0 ACP, palmACP[*c*]) was chosen as the *Target product* in the UP Finder. Fig. 4 shows the output results. A total of 30 metabolic reactions, involving 6 genes (*accABCD*, *fabD*, *fabF*, *fabG*, *fabZ*, *fabI*) were identified as the key pathways/genes for palmitoyl-ACP overproduction. 28 out of the 30 reactions are catalyzed by fatty acid synthases in *Synechocystis* sp. PCC 6803, in which 4 key genes (*fabF*, *fabG*, *fabZ*, *fabI*) are involved. The reactions were ordered by their associated *Ratio* values, and the different *Ratio* values of the 30 identified up-regulated pathways represent different up-regulation levels to achieve the same *Yield* (theoretical maximum yield). Thus, the higher *Ratio* values indicated the higher demands of metabolic flux for up-regulation toward palmitoyl-ACP overproduction. On the other hand, the completed fatty acid synthesis pathways of the constraint-based model enabled the detailing of fluxes for each single reaction in multiple-repeated fatty acid biosynthesis. It was found that the reactions with same *Ratio* values reflected the similar level of metabolic flux for going through, which might be used as a quantitative standard for identifying metabolic modules in complex metabolic pathways.

Similar to the finding of Example 1, it was also found that the higher preferred gene targets presented closer metabolic distance to palmitoyl-ACP. Compared with reported key genes regarding to fatty acyl-ACP overproduction in the literatures (Liu et al., 2011), results identified by the UP Finder show 100% identity.

## 4 Discussion

In this study, a modeling software package named UP Finder was developed based on the COBRA toolbox in MATLAB. It facilitated the rapid identification of gene overexpression strategies for the metabolic engineering of targeted overproduction. Gene overexpression targets can be rationally determined by a quantitative evaluation procedure. Development of this interfacial software package was designed to provide the “one-click” convenience, and to facilitate the access for potential users without specific biochemistry and programming backgrounds. By taking advantage of standardized format of SBML models, UP Finder provided broad access for analyzing various targeted products in different microorganisms. Unlike OptForce and FSEOF, UP Finder specifically identified gene targets that were highly related to overproduction rather than all potential important targets. The UPA method used in the UP Finder investigated product yields for each single potential up-regulated pathway by constraining their fluxes with *flux*_*opt*_, which evaluated their contribution toward theoretical maximum yield and enabled to pinpoint the results as yield/overproduction related. Although some key upstream pathways, such as glycolysis play an important role in improving target product yields when combining with downstream pathway enhancements, the sole overexpression of these genes might not directly contribute to the overproduction.

The quantitative evaluation that combining *Ratio* and *Yield* parameters improved the relevance of identified pathways for directly leading to the overproduction. In the UP Finder, gene overexpression targets were first identified as up-regulated pathways (the reaction with *Ratio* > 0) through a flux comparison between the theoretical maximum yield model and the wild-type model. By using *flux*_*opt*_ as a constraint, the contribution of each single identified up-regulated pathway on targeted overproduction was quantitatively evaluated based on the yield simulation. The restriction of *Yield* > 0 was used to exclude the reactions that are indirectly related to the overproduction, such as the pathways in central carbon metabolism. Thus, the UP Finder provided a quantitative procedure for identifying the key pathways toward overproduction. In addition, it was found in the examples that pathways with higher *Ratio* values showed closer metabolic distance to the targeted product. Since the greater *Ratio* value (up-regulation level) indicates the higher demands of metabolic flux for up-regulation to approach the theoretical maximum yield, overexpression of the particular gene would contribute more to the overproduction of targeted products. Therefore, results from the UP Finder not only presented all the key pathway/gene targets related to the overproduction, but the ranking of the outputs with their associated *Ratio* values also reflected the preference for considering overexpression strategies in pathway design.

On the other hand, the *Ratio* parameter can be used for identifying functional metabolic modules. In the analysis examples, it was found that pathways with similar level of *Ratio* values presented the adjacent locations with regard to metabolic functions. For example, in Example 1 (Fig. 3), identified key pathways can be divided into 3 metabolic modules by their *Ratio* levels, including the initial MEP pathway synthesis module (*dxs*), the isoprenoid unit synthesis module (*dxr*, *ispD*, *ispE*, *ispF*, *ispG*, *ispH*), and the FPP synthesis module (*idi*, *ispA*). In Example 2 (Fig. 4), pathways with similar chain length of fatty acid synthesis also showed the similar levels on their *Ratio* values. In metabolic engineering of secondary metabolites and complex metabolic pathways, the imbalance of metabolic flux is a critical limiting factor for reaching high product yields. To coordinate the metabolic imbalance, engineering of module-based metabolic optimization has been regarded as a promising strategy for optimizing product yields (Ajikumar et al., 2010, Xu et al., 2013, Yadav et al., 2012, Zhao et al., 2013). Therefore, by taking advantage of the *Ratio* parameter, outputs of the UP Finder could also provide a quantitative basis for identifying functional metabolic modules in developing module-based optimization strategies.

In this version of the UP Finder, not all metabolites listed in the reconstructed model will have valid results. It is usually good for analyzing terminal metabolites, such as secondary metabolites. For metabolites with considerable degradation pathways, it may not have valid results because the up-regulated flux would be further consumed by the degradation without accumulation. Since the evaluation process only works for single pathway, the UP Finder does not provide the best combination of overexpression yet in this version. To achieve accurate prediction and high identity with experimental verification, a high quality of metabolic network reconstruction is necessary. Some current SBML models such as *E. coli* (iJO1366.xml), and *Synechocystis* sp. PCC 6803 (iJN678.xml) have been tested with valid outputs in this UP Finder. Analysis examples demonstrated that the UP Finder is feasible to analyze gene overexpression targets for overproducing secondary metabolites and complex metabolic pathways, such as fatty acid biosynthesis. Given the decreasing cost of DNA synthesis, fast strain development for overproducing targeted products is becoming possible based on the large-scale DNA synthesis. Therefore, a user-friendly interfacial modeling tool that provides rapid pathway design would play an important role in the era of synthetic biology (Gibson, 2014, Kosuri and Church, 2014, Wang et al., 2011).

## 5 Conclusions

In this study, a modeling tool named UP Finder was developed based on the COBRA toolbox. It facilitated the rapid identification of gene overexpression strategies to assist pathway design in metabolic engineering of targeted overproduction. Gene targets with highly related to overproduction were determined by a quantitative evaluation procedure. The graphical user interface of the UP Finder provided easier access for analyzing various targeted products in different microorganisms. Analysis examples for overproducing lycopene precursor and fatty acyl-ACP by the UP Finder showed high degree of agreement with the reported key genes in the literatures.

## Footnotes

Appendix A Supplementary data associated with this article can be found in the online version at doi:10.1016/j.meteno.2017.08.001.
