# Supplementary material for: UP Finder: A COBRA toolbox extension for identifying gene overexpression strategies for targeted overproduction
Source: Metab Eng Commun. 2017 Aug 16;5:54–9. doi: 10.1016/j.meteno.2017.08.001 (PMC5699525; doi:10.1016/j.meteno.2017.08.001)
Supplement: Supplementary file 1 — Supplementary material [file mmc1.docx]

Supplementary Material

**UP Finder: A graphical user interface for identifying gene overexpression strategies for targeted overproduction**

Xi Wang, Liang Yu, Shulin Chen*

Department of Biological Systems Engineering, Washington State University, Pullman, WA 99164, USA

*Corresponding author. E-mail: [chens@wsu.edu](mailto:chens@wsu.edu). Phone: +1 (509)-335-3743. Fax: +1 (509)-335-2722.

**Table S1** The description of abbreviated reaction names shown in the UP Finder results ^1,2^

| **Abbreviation in Fig. 3** | **Reaction name** |
| --- | --- |
| GRTT | farnesyl diphosphate synthase |
| DMATT | geranyltranstransferase |
| IPDDI | isopentenyl-diphosphate D-isomerase |
| MEPCT | 2-C-methyl-D-erythritol 4-phosphate cytidylyltransferase |
| MECDPS | 2-C-methyl-D-erythritol 2,4-cyclodiphosphate synthase |
| MECDPDH5 | 2C-methyl-D-erythritol 2,4 cyclodiphosphate dehydratase |
| IPDPS | 1-hydroxy-2-methyl-2-(E)-butenyl 4-diphosphate reductase |
| DXPRIi | 1-deoxy-D-xylulose reductoisomerase |
| CDPMEK | 4-(cytidine 5'-diphospho)-2-C-methyl-D-erythritol kinase |
| DXPS | 1-deoxy-D-xylulose-5-phosphate synthase |
| **Abbreviation in Fig. 4** | **Reaction name** |
| EAR160y | enoyl-[acyl-carrier-protein] reductase (NADPH) (n-C16:0) |
| EAR140y | enoyl-[acyl-carrier-protein] reductase (NADPH) (n-C14:0) |
| 3OAS160 | 3-oxoacyl-[acyl-carrier-protein] synthase (n-C16:0) |
| 3HAD160 | 3-hydroxyacyl-[acyl-carrier-protein] dehydratase (n-C16:0) |
| 3HAD140 | 3-hydroxyacyl-[acyl-carrier-protein] dehydratase (n-C14:0) |
| 3OAR160 | 3-oxoacyl-[acyl-carrier-protein] reductase (n-C16:0) |
| 3OAR120 | 3-oxoacyl-[acyl-carrier-protein] reductase (n-C12:0) |
| EAR120y | enoyl-[acyl-carrier-protein] reductase (NADPH) (n-C12:0) |
| EAR100y | enoyl-[acyl-carrier-protein] reductase (NADPH) (n-C10:0) |
| 3OAS140 | 3-oxoacyl-[acyl-carrier-protein] synthase (n-C14:0) |
| 3OAS120 | 3-oxoacyl-[acyl-carrier-protein] synthase (n-C12:0) |
| 3OAR140 | 3-oxoacyl-[acyl-carrier-protein] reductase (n-C14:0) |
| 3HAD120 | 3-hydroxyacyl-[acyl-carrier-protein] dehydratase (n-C12:0) |
| 3OAR80 | 3-oxoacyl-[acyl-carrier-protein] reductase (n-C8:0) |
| 3OAR60 | 3-oxoacyl-[acyl-carrier-protein] reductase (n-C6:0) |
| 3OAR40 | 3-oxoacyl-[acyl-carrier-protein] reductase (n-C4:0) |
| 3OAR100 | 3-oxoacyl-[acyl-carrier-protein] reductase (n-C10:0) |
| KAS15 | beta-ketoacyl-ACP synthase (2) |
| EAR80y | enoyl-[acyl-carrier-protein] reductase (NADPH) (n-C8:0) |
| EAR60y | enoyl-[acyl-carrier-protein] reductase (NADPH) (n-C6:0) |
| EAR40y | enoyl-[acyl-carrier-protein] reductase (NADPH) (n-C4:0) |
| 3OAS80 | 3-oxoacyl-[acyl-carrier-protein] synthase (n-C8:0) |
| 3OAS60 | 3-oxoacyl-[acyl-carrier-protein] synthase (n-C6:0) |
| 3OAS100 | 3-oxoacyl-[acyl-carrier-protein] synthase (n-C10:0) |
| 3HAD80 | 3-hydroxyacyl-[acyl-carrier-protein] dehydratase (n-C8:0) |
| 3HAD60 | 3-hydroxyacyl-[acyl-carrier-protein] dehydratase (n-C6:0) |
| 3HAD40 | 3-hydroxyacyl-[acyl-carrier-protein] dehydratase (n-C4:0) |
| 3HAD100 | 3-hydroxyacyl-[acyl-carrier-protein] dehydratase (n-C10:0) |
| MCOATA | Malonyl-CoA-ACP transacylase |
| ACCOACr | acetyl-CoA carboxylase, reversible reaction |

## **Table S2** The description of abbreviated metabolite names shown in the UP Finder results ^1,2^

| **Abbreviation** | **Metabolite name** |
| --- | --- |
| 2me4p[c] | 2-C-methyl-D-erythritol 4-phosphate (MEP) |
| 2mecdp[c] | 2-C-methyl-D-erythritol 2,4-cyclodiphosphate (MEC) |
| 2p4c2me[c] | 4-diphosphocytidyl-2C-methyl-D-erythritol-2-phosphate (CDP-MEP) |
| 3haACP[c] | (3R)-3-Hydroxyacyl-ACP |
| 3hddecACP[c] | (R)-3-Hydroxydodecanoyl-ACP |
| 3hdecACP[c] | (R)-3-Hydroxydecanoyl-ACP |
| 3hhexACP[c] | (R)-3-Hydroxyhexanoyl-ACP |
| 3hmrsACP[c] | (R)-3-Hydroxytetradecanoyl-ACP |
| 3hoctACP[c] | (R)-3-Hydroxyoctanoyl-ACP |
| 3hpalmACP[c] | R-3-hydroxypalmitoyl-ACP |
| 3oddecACP[c] | 3-Oxododecanoyl-ACP |
| 3odecACP[c] | 3-Oxodecanoyl-ACP |
| 3ohexACP[c] | 3-Oxohexanoyl-ACP |
| 3omrsACP[c] | 3-Oxotetradecanoyl-ACP |
| 3ooctACP[c] | 3-Oxooctanoyl-ACP |
| 3opalmACP[c] | 3-Oxohexadecanoyl-ACP |
| 4c2me[c] | 4-diphosphocytidyl-2-C-methyl-D-erythritol (CDP-ME) |
| accoa[c] | Acetyl-CoA |
| ACP[c] | Acyl carrier protein |
| actACP[c] | Acetoacetyl-ACP |
| adp[c] | ADP |
| atp[c] | ATP |
| but2eACP[c] | But-2-enoyl-ACP |
| butACP[c] | Butyryl-ACP (n-C4:0 ACP) |
| cmp[c] | CMP |
| co2[c] | CO_2_ |
| coa[c] | Coenzyme A |
| ctp[c] | CTP |
| dcaACP[c] | Decanoyl-ACP (n-C10:0 ACP) |
| ddcaACP[c] | Dodecanoyl-ACP (n-C12:0 ACP) |
| dmpp[c] | Dimethylallyl diphosphate (DMAPP) |
| dxyl5p[c] | 1-deoxy-D-xylulose 5-phosphate (DXP) |
| flxr[c] | Flavodoxin reduced |
| flxso[c] | Flavodoxin semi oxidized |
| frdp[c] | Farnesyl pyrophosphate (FPP) |
| g3p[c] | Glyceraldehyde 3-phosphate (GAP) |
| grdp[c] | Geranyl pyrophosphate (GPP) |
| h[c] | H^+^ |
| h2mb4p[c] | (E)-4-hydroxy-3-methylbut-2-enyl-diphosphate (HMBPP) |
| h2o[c] | H_2_O |
| hco3[c] | Bicarbonate |
| hexACP[c] | Hexanoyl-ACP (n-C6:0 ACP) |
| ipdp[c] | Isopentenyl diphosphate (IPP) |
| malACP[c] | Malonyl-ACP |
| malcoa[c] | Malonyl-CoA |
| myrsACP[c] | Myristoyl-ACP (n-C14:0 ACP) |
| nad[c] | Nicotinamide adenine dinucleotide (NAD^+^) |
| nadh[c] | Nicotinamide adenine dinucleotide - reduced (NADH) |
| nadp[c] | Nicotinamide adenine dinucleotide phosphate (NADP^+^) |
| nadph[c] | Nicotinamide adenine dinucleotide phosphate - reduced (NADPH) |
| ocACP[c] | Octanoyl-ACP (n-C8:0 ACP) |
| palmACP[c] | Palmitoyl-ACP (n-C16:0 ACP) |
| pi[c] | Phosphate |
| ppi[c] | Diphosphate |
| pyr[c] | Pyruvate |
| tddec2eACP[c] | trans-Dodec-2-enoyl-ACP |
| tddec2eACP[c] | trans-Dodec-2-enoyl-ACP |
| tdec2eACP[c] | trans-Dec-2-enoyl-ACP |
| thex2eACP[c] | trans-Hex-2-enoyl-ACP |
| tmrs2eACP[c] | trans-Tetradec-2-enoyl-ACP |
| toct2eACP[c] | trans-Oct-2-enoyl-ACP |
| tpalm2eACP[c] | trans-Hexadec-2-enoyl-ACP |

**References**

1 Orth, J. D. *et al.* A comprehensive genome-scale reconstruction of *Escherichia coli* metabolism—2011. *Mol. Syst. Biol.* **7** (2011).

2 Nogales, J., Gudmundsson, S., Knight, E. M., Palsson, B. O. & Thiele, I. Detailing the optimality of photosynthesis in cyanobacteria through systems biology analysis. *Proc. Natl. Acad. Sci. U. S. A.* **109**, 2678–2683 (2012).
